# A phase II trial of a biweekly combination of paclitaxel and gemcitabine in metastatic breast cancer

**DOI:** 10.1186/1471-2407-6-137

**Published:** 2006-05-24

**Authors:** Silverio Tomao, Adriana Romiti, Federica Tomao, Marisa Di Seri, Giuliana Caprio, Gian Paolo Spinelli, Edmondo Terzoli, Luigi Frati

**Affiliations:** 1 Department of Clinical Oncology, "Regina Elena" Cancer Institute of Rome, Via Chianesi, 53, 00100 Rome, Italy; 2 Department of Experimental Medicine and Pathology, University of Rome "La Sapienza", Viale Regina Elena 324, 00161 Rome, Italy; 3 Department of Medical Oncology, S.Andrea General Hospital, Via di Grottarossa, Rome, Italy

## Abstract

**Background:**

Many emerging new drugs have recently been trialled for treatment of early and advanced breast cancer. Among these new agents paclitaxel and gemcitabine play a crucial role, mostly in patients with relapsed and metastatic disease after failure of chemotherapy with antracyclines.

**Methods:**

A phase II study was started in order to evaluate the activity and toxicity of a combination of paclitaxel and gemcitabine in a biweekly schedule on metastatic breast cancer patients previously treated with antracyclines.

**Results:**

Twenty-five patients received paclitaxel (150 mg/mq) by 3-hours infusion, followed by gemcitabine (2000 mg/mq) given as a 60 min i.v. infusion (day 1–14) for a maximum of eight cycles. In all patients treatment was evaluated for toxicity and efficacy; four patients (16%) achieved a complete response, 12 (48%) a partial response giving an overall objective response rate of 64%. Stable disease was documented in 5 patients (20%) and progressive disease occurred in 4 patients (16%).

**Conclusion:**

The schedule of treatment was safe and tolerable from a haematological and non-haematological point of view. These data confirm that the combination of gemcitabine and paclitaxel on a biweekly basis is an effective and well-tolerated regimen in breast cancer patients with prior therapeutic exposure to antracyclines.

## Background

Despite the availability of new, active drugs, metastatic breast cancer (MBC) remains an incurable disease, and the treatment of it is still controversial. In fact, the best treatment for patients pretreated with antracyclines, a subset very common in clinical practice, is hotly debated, given the extensive use of doxorubicine and epirubicine in metastatic, adjuvant and neoadjuvant settings.

Nevertheless, today the treatment of MBC is a rational choice, as different studies have shown that the use of polichemotherapy in this subset of patients appears to improve long term remission, relapse-free survival and overall survival [1-4].

In recent years, novel drugs have emerged as important agents in the treatment of MBC patients, because of their safety and efficacy in generating symptom relief, in reducing disease progression and in prolonging survival.

Among these new agents, taxanes have become the standard therapy in patients pretreated with antracyclines. In this subset of patients paclitaxel as a single agent has generated response rates ranging from 6% to 48% [5,6] and these results improved when the drug was used as first line treatment in metastatic disease[7]. Actually a number of newer cytotoxic agents have been introduced in clinical trials to evaluate novel, safe and active taxane-based combinations in the treatment of MBC, extensively pretreated with antracyclines.

Gemcitabine is a cytidine nucleoside analogue with proven activity in advanced breast cancer.

In previously treated MBC patients it has produced response rates ranging from 12% to 29%, and it was tolerated satisfactorily [8,9], while in first line schedules the response rate reported was 14–37% [10].

The paclitaxel and gemcitabine combination is justified by their different mechanism of action and by the lack of overlapping toxicities. In MBC this combination has been evaluated in phase II studies using a three -weekly schedule of treatment with paclitaxel given at 175 mg/mq on day 1 and gemcitabine given at 1000–1250 mg/mq on days 1,8, showing an interesting response rate ranging from 45% to 55% [11,12]. Recently an interim analysis of a large phase III study demonstrated that the combination of paclitaxel plus gemcitabine as first-line treatment was more efficacious than paclitaxel alone in MBC, according to the different clinical variables considered (progression-free survival, response rate, pain relief and QOL)[13].

In a phase II study the combination of paclitaxel and gemcitabine was explored in a biweekly schedule by Colomer et al. in 1998 in a subset of patients who had not received prior treatment for MBC [14]. In this trial the response rate was impressive with an overall response of 69% (24% CR), and was well tolerated.

The same authors have recently updated these data in untreated MBC patients, with an overall response rate of 71% (26% CR). Moreover, in the same study it was demonstrated that the efficacy of this schedule could be reduced by elevated levels of HER2 [15].

According to these results a phase II study was started to evaluate the efficacy and tolerability of a biweekly schedule of paclitaxel and gemcitabine in MBC patients pretreated with antracyclines.

## Methods

### Eligibility

To be elegible for the study, patients were required to have histologically confirmed breast cancer, metastatic or locally advanced disease, bidimensionally measurable lesions, performance status ≥ 70%, age 18–75 ys, adequate bone marrow, hepatic and renal function (neutrophil count ≥ 1500/μL, platelets ≥ 100,000 μL, hemoglobin ≥ 10 g/L, bilirubin ≤ 2 mg/dl, creatinine 1.5 ≤ mg/dl, and alanine/aspartate amino transferase level ≤ 3 times above normal). Prior chemotherapy, excluding gemcitabine and taxanes, radiotherapy and endocrine-therapy were permitted. All the patients were required to have received prior chemotherapy with antracyclines, in neoadjuvant, adjuvant and metastatic setting. Patients were excluded from the study in cases of: brain metastases, peripheral neuropathy and vasculopathy, bone metastases as the only site of disease, history of active cardiac disease, previous malignant neoplasia, pregnancy and breast feeding, any antineoplastic treatment within 8 weeks of entering the study

.

### Treatment plan

Study treatment consisted of the infusion of gemcitabine and paclitaxel according to a biweekly schedule. Patients received paclitaxel (150 mg/mq) in 3-hours infusion, followed by gemcitabine (2000 mg/mq) given as a 60 min i.v. infusion. Patients received standard premedication with i.v. dexamethasone (20 mg) and antiemetic treatment 1 hour before the start of therapy with paclitaxel, plus orphenadrine and cimetidine.

Patients were scheduled to receive a maximum of 8 cycles and chemotherapy was stopped in case of progression, patient refusal and unacceptable toxicity. Patients who had received at least one course of chemotherapy were evaluated for toxicity and at least two courses for efficacy. The toxicity and activity of the schedule were evaluated according to the WHO criteria. [16]. All the measurable lesions were evaluated at baseline and after every two courses in order to document any response, stable disease or progressive disease.

Complete response was defined as the disappearance of all measurable lesions, partial response as the decrease of more than 50% of the sum of the products of all the measured lesions with no occurrence of new lesions, stable disease as a reduction ranging from 25% to 50% of the sum of the products of all the measured lesions with no occurrence of new lesions and progressive disease as an increase > 25% of the sum of the products of all the measured lesions or the occurrence of new lesions.

All the patients were required to give written informed consent before the start of treatment and the study was conducted according to the approval of the local ethical board (Azienda Policlinico Umberto I – Ethical Committee)

## Results

### Patients characteristics

Twenty-five consecutive patients with metastatic breast cancer and measurable disease were recruited by the Department of Experimental Medicine La Sapienza University of Rome in order to assess the tolerability and efficacy of a biweekly schedule of paclitaxel and gemcitabine. All the patients had received prior chemotherapy with antracyclines in adjuvant and non adjuvant settings and sixteen of them have been treated for metastatic disease. All the patients were evaluated for toxicity and efficacy. The main characteristic of the patients are shown in table 1.

The median age was 51 years and the youngest patient enrolled was 39 years old. The majority of patients presented a WHO performance status of 0 (80%). All the patients had received prior treatment with antracyclines (44 % in adjuvant setting). At diagnosis lung and liver involvement was detected in 15 patients (60% with dominant visceral disease).

### Efficacy

A total of 148 cycles were given in an output basis with a median of seven per patient.

Four patients (16%) achieved a complete response, 12 (48%) a partial response, with an overall objective response rate of 64%. Stable disease was documented in 5 patients (20%) while progressive disease occurred in 4 patients(16%).

After an average follow-up of 18 months, the median duration of response in the subset of responders was 11.5 months with twelve patients alive.

Table 2 shows the response rates observed in the study.

### Toxicity

Toxicity data were available for all the patients recruited. Treatment was well tolerated in almost all the patients with infrequent occurrence of 3–4 grade toxicity.

The most frequent toxicity was haematological : two patients experienced grade 4 neutropenia and one grade 4 thrombocytopenia; grade 3 neutropenia occurred in 24% of patients; thrombocytopenia in 16% and anemia in 12%. No febrile neutropenia was observed and none of the patients received platelet transfusion. No toxic death or hospitalisation occurred.

Grade 2 peripheral neuropathy was frequent, mainly as grade 1–2 (38% of cases).

Complete alopecia occurred in almost all the patients treated.

Dose reduction or delays were necessary in less than 10% of chemotherapy cycles.

A summary of the toxicity data is reported in Table 3.

## Discussion

Although advanced and relapsing breast cancer is generally considered an incurable disease, many opportunities have been explored in recent years to achieve an efficient palliation with the combined use of chemotherapy, radiotherapy, endocrine therapy and supportive treatment Systemic chemotherapy must be considered the treatment of choice in this subset of patients, in particular when endocrine resistance occurs. Antracyclines still constitute the cornerstone of chemotherapeutic approaches in advanced breast cancer, while the problem of the best schedule of treatment when an antracycline resistance occurs still has to be resolved.

In monochemotherapy, paclitaxel has shown interesting activity in doxorubicin-refractory metastatic breast cancer (28–48% response rate) and many phase II and III trials have investigated its optimal combination with other anticancer drugs. Among the new agents investigated in the treatment of advanced breast cancer, gemcitabine has demonstrated unexpected activity with response rates ranging from 15% to 40% when used as single agent in first- and second-line therapy. Moreover, the choice of this drug in the treatment od relapsing and metastatic breast cancer is conditioned by its toxicity profile. According to these data, the combination of paclitaxel and gemcitabine in patients with advanced and metastatic breast cancer and pretreated with antracyclines has recently been investigated in some trials. Nevertheless, the best schedule of treatment with a combination of the two drugs is still under investigation (weekly, biweekly, etc...), and other studies are necessary to address this question, in particular when gemcitabine and paclitaxel are used in triplet schedules, in combination with other traditional or innovative drugs.

For this reason we investigated the toxicity and the clinical activity of the combination of gemcitabine and paclitaxel according to the schedule reported by Colomer [14] with a biweekly infusion of the two drugs in untreated patients with metastatic breast cancer.

In our study the dose of gemcitabine was lower than that reported by Colomer (2000 mg/mq vs 2500 mg/mq), using gemcitabine with precaution in our group of heavily pretreated patients.

In spite of this reduction the response rate in our experience was surprisingly high, although the patients had received two and more lines of treatment in the past. Moreover, only sixteen patients experienced progressive disease during the treatment, confirming the activity of the combination of gemcitabine and paclitaxel in the palliative therapy of metastatic breast cancer.

The favourable toxicity profile of this schedule was confirmed by the satisfactory results of other studies, and led to testing of this schedule in combination with new drugs with different biological activity (trastuzumab, tyrosine kinase and VEGF inhibitors). Moreover, these data suggest that the introduction of the combination as first line treatment of MBC should be explored more extensively only after its activity as second line treatment in better understood and the best schedule of infusion has been identified.

Thus, some questions arise from this and other studies. With this novel combination which schedule of treatment in the best, weekly or biweekly? With the gemcitabine/paclitaxel combination which is the right dose and is intensification or acceleration of the dose possibility? Is this combination a valid alternative in first-line treatment of patients not candidate to receive chemotherapy with antracyclines for clinical and biological reasons?

## Conclusion

The study reported in this paper presents an evident methodological limit: the number of patients enrolled; in spite of the strong evidence in literature activity of the combination of gemcitabine and paclitaxel in metastatic breast cancer, the papers recently published in this field present the same limit.

This evidence data support further testing of this combination in a larger randomized phase III clinical trial.

Unfortunately because there is not a standard in the choice of the best chemotherapeutic treatment of metastatic breast cancer resistant to anthracyclines, it is difficult to design a randomized trial to compare this novel association (on weekly or biweekly schedule) with a control scheme of treatment. Moreover this clinical limitation is complicated by the extensive use of taxanes in first line chemotherapy of breast cancer and therefore the gold standard after anthracyclines/taxanes failure is fat to be identified.

Nevertheless it is evident that there is a strong indication to start also with a III phase trial to compare the weekly with the biweekly schedule ; moreover it will be very interesting to evaluate the role of emerging prognostic factors (e.g. HER2/neu gene amplification, VEGF, e-cadherin...) in patients treated with this association with the aim to select chemosensitive patients.

## Competing interests

The author(s) declare that they have no competing interests.

## Authors' contributions

ST designed the study, performed the statistical analysis, followed the patients, drafted the manuscript and coordinated the submission. AR, MDS, GC, ET followed the patients. FT, GPS, LF performed the statistical analysis, revised the literature, followed the patients and involved in the final revision of the manuscript. All authors read and approved the final manuscript.

## Pre-publication history

The pre-publication history for this paper can be accessed here:



## Figures and Tables

**Table 1:** Characteristics of patients (n.25)

**Characteristic**	**n(%)**
Age (years)	
*Median*	51
*Range*	39–69
	
P. S 0–1	20(80%)
P. S 2	5 (20%)
	
Histology	
Infiltrating ductal ca	19 (76)
Infiltrating lobular ca	6 (14)
	
Estrogen status	
ER+	14 (56)
ER -	7 (28)
Unknown	4 (16)
	
Dominant sites of metastases	
Nodes	10(40)
Soft tissues	2(8)
Bone	5(20)
Liver	8(32)
Lung	7(28)
	
Pretreatment with antracyclines	
Adjuvant CT	11(44)
Not adjuvant CT	14(56)

**Table 2:** Response rates

**Response**	**No of patients (%)**
Complete response	4 (16)
Partial response	12(48)
Overall response	16(64)
Stable disease	5 (20)
Progressive disease	4(16)

**Table 3:** Hematological and non-hematological toxicity(% patients)

**Toxicity**	**Grade 3**	**Grade 4**
Anaemia	12	-
Neutropenia	24	8
Trombocytopenia	16	4
Neuropathy	-	-
Mucositis	-	-
